# Dental Teachings

**Published:** 1864-08

**Authors:** Wm. H. Atkinson


Dental Teachings.—*Read before the New York Society of Dental Surgeons—By Wm. H. Atkinson, M. D.*—Every sort of teaching has its advocate, who affirms with great confidence that his particular school or method is *the* one upon which to rely for rapid and sure advance.

All schools in medicine, surgery and dentistry, so far as I know, are in the habit of requiring a nominal or real pupilage, measured by *time* spent, or said to be spent, in what is called "study of the principles and practice" pertaining to the particular field of investigation.

The best way to learn what school or course most facilitates acquisition, is a close survey and sharp scrutiny of those in practice of medicine, surgery and dentistry.

And let me ask who are the *men* in each department? Are they from the schools replete with erudition? or are they not rather the so-called rough, uneducated, strong mind from the country?

And why is this so? Is it because schools are unnecessary, and a hindrance to true progress? or is it not rather that the practice of those holding the positions of teachers in depending upon obsolete formularies to lighten the labor of their teaching, that so many self-taught men are the superiors of those of equal talent and energy, who have sedulously passed the ordeals of the schools?

Mere memorizing of formularies will never illuminate the understanding. It requires that our queries be answered to our individual apprehension, to constitute them bases upon which we can depend in the days of our need in practice, when we shall have no teacher or book at hand upon which to shirk the responsibilities we have taken upon us, when announcing ourselves doctors, surgeons, or dentists. I, too, have my pet method and means of teaching, and that is to hold familiar, and laborious, and sometimes humiliating conversations with my pupils, on all the subjects which engage our attention; and thus demonstrate as we go, so far as we may, all the doctrines and practices I advocate.

Blind adherence to precedent is probably the greatest barrier in the way of dental teaching. He who would do his class the greatest good in the shortest space must begin "de novo," and really sacrifice himself to their benefit. He must not only feel that he knows but little about the matters desirable to teach, but also be willing that he so appear to his earnest fellow-seekers after truth!

In view of this statement, is it any wonder why so many graduates of the various schools are so vitally deficient in diagnostic and manipulative ability?

Who among us all is in the least fit to teach, if what I say is true?

Isolation of chairs, schools, and investigators may facilitate discovery, in special directions of laborious detail, but fusion of chairs, and schools, and close fraternity of labor and mutual interest among investigators, can alone establish knowledge in associate or codified and useful forms!

What, then, shall be done for dentistry in view of these apparently insurmountable obstacles? The best course to pursue for this and all time is, to make the main object of teaching and learning to consist in the attainments of knowledge by both teacher and pupil! irrespective of who or what the pupil may be who applies to us to enter our classes; making no unnecessary conditions, nor opening any door by which unqualified persons can by possibility attain the indorsement for acquirements they do not possess!

My ripest judgment tells me this end can in no known way be so readily attained as to make all our teachings consist in conversations, and lively discussions, and clinical demonstrations, in which all are required to enter with child-like earnestness and fraternity.

Just so soon as each mind is set fully at liberty, by assuring it that it is safe to allow others to know how deplorably deficient it feels itself to be in matters of really demonstrated knowledges, we shall have such a oneness of aspiration as to make it next to impossible to hold us back from the attainment of demonstration to our inquiries!

The real investigator in matters of science is above or below, whichever you please, the petty desire to domineer over other men's minds and pursuits, and willingly accepts and adopts whatever of demonstration he is favored with by his fellows; nor is he slow to give due credit and acknowledgment to all such noble helps against the host of ignorance with which we all have to contend.

All that dentistry is, it owes to the eminently demonstrative character of its nature, and an almost untrodden field of its investigators. And although we can never abrogate the necessity of anatomical principles and facts, yet where these have not been understood nor explored, we may not look to be benefited by our predecessors

If we are to have "Dental Teachings" culminate in a well-digested code of "dental institues," it behooves each one to communicate all the knowledge he possesses to the common stock, that it may become indeed common; and thus serve as a substratum upon which to stand to make yet further researches into the elements constituting a science; which, when attained, is sure to make our most brilliant present knowledge appear as but letters in the alphabet of the coming histology, physiology, and pathology of dental tissues, and teachings of doctrines how to prevent and how best to remedy the aberrations from healthy function to which these are now liable!—*Dental Cosmas.*

*New York, May* 16*th*, 1864.

